# Case Report: Severe back pain, epigastric distress and refractory nausea; an unusual presentation of mediastinal bronchogenic cyst

**DOI:** 10.12688/f1000research.15128.1

**Published:** 2018-06-28

**Authors:** Saeed Ali, Abdul Rauf, Ling Bing Meng, Zeeshan Sattar, Sana Hussain, Umair Majeed

**Affiliations:** 1 Internal Medicine Residency, Florida Hospital Orlando, Orlando, FL, 32803, USA; 2 Khyber Teaching Hospital, Peshawar, Pakistan

**Keywords:** bronchogenic, cyst, back, pain

## Abstract

**Background:** Bronchogenic cysts are congenital malformations from abnormal budding of embryonic foregut and tracheobronchial tree. We present a case of bronchogenic cyst with severe back pain, epigastric distress and refractory nausea and vomiting.

**Case Presentation:** A 44-year-old Hispanic female presented with a 3-week history of recurrent sharp interscapular pain radiating to epigastrium with refractory nausea and vomiting. She underwent cholecystectomy 2-years ago. Computed tomography (CT) abdomen at that time showed a subcarinal mass measuring 5.4 X 5.0 cm. Subsequent endoscopic ultrasound diagnosed it as a bronchogenic cyst. Endobronchial ultrasound (EBUS) guided aspiration resulted in incomplete drainage and she was discharged after partial improvement. Current physical examination showed tachycardia and tachypnea with labs showing leukocytosis, elevated inflammatory markers, and hypokalemic metabolic alkalosis. CT chest showed an increased size of the bronchogenic cyst (9.64 X 7.7 cm) suggestive of possible partial cyst rupture or infected cyst. X-ray esophagram ruled out esophageal compression or contrast extravasation. Patient’s symptoms were refractory to conservative management. The patient ultimately underwent right thoracotomy with cyst excision that resulted in complete resolution of symptoms.

**Conclusion:** Bronchogenic cysts are the most common primary cysts of mediastinum with the prevalence of 6%. The most common symptoms are chest pain, dyspnea, cough, and stridor. Diagnosis is made by chest X-Ray and CT chest. Magnetic resonance imaging chest and EBUS are more sensitive and specific. Symptomatic cysts should be resected unless surgical risks are high. Asymptomatic cysts in younger patients should be removed due to low surgical risk and potential late complications. Watchful waiting has been recommended for asymptomatic adults or high-risk patients.

This case presents mediastinal bronchogenic cyst as a cause of back, nausea and refractory vomiting. Immediate surgical excision in such cases should be attempted, which will lead to resolution of symptoms and avoidance of complications.

## Introduction

Bronchogenic cysts are congenital malformations of the bronchial tree. They result from the anomalous development of ventral foregut and tracheobronchial tree ^1^. They can present as a mediastinal mass that may enlarge and cause local compression. They are the most common primary cysts of the mediastinum with a prevalence of 6% ^2^. Approximately 79% of cysts are located in the middle mediastinum, 17% in posterior mediastinum and 3% in the anterior mediastinum ^3^.

Almost 75% of bronchogenic cysts are asymptomatic. Symptoms vary with age at presentation and with size and location of the cyst. The common symptoms include chest pain (22%), dyspnea (12%), cough (7%), stridor (7%) and respiratory compromise due to tracheal/bronchial compression (10%). Unusual manifestations are dysphagia (1%), pneumothorax (1%), and superior vena cava syndrome (1%) ^3^.

Diagnosis is made by chest X-Ray and computed tomography (CT) chest although magnetic resonance imaging (MRI) chest and endobronchial ultrasound are highly sensitive and specific ^4^. MRI chest can provide additional information about the consistency and nature of the cyst depending upon the presence of proteinous contents in the fluid. In general, bronchogenic cyst appears hypo-intense on T1-weighed images and hyper-intense on T2-weighed images ^5^. Endoscopic ultrasound (EUS) is a relatively invasive procedure for the diagnosis of the bronchogenic cyst.

Treatment options depend on patient’s age and symptoms. Symptomatic bronchogenic cyst are managed surgically with resection, Endobronchial ultrasound (EBUS) guided aspiration, and video-assisted thoracoscopic surgery being a minimally invasive procedure ^6^. Thoracotomy is performed for difficult cases. Asymptomatic cysts in younger patients should be removed due to low surgical risk and potential late complications such as infection, hemorrhage or neoplasia. Watchful waiting has been recommended for asymptomatic adults or high-risk patients. Percutaneous drainage or alcohol ablation has been performed in selected cases ^4^. We present a case of a mediastinal bronchogenic cyst in a 44-year-old female presenting in the form of severe back pain, epigastric distress and nausea.

## Case report

**Figure 1.**
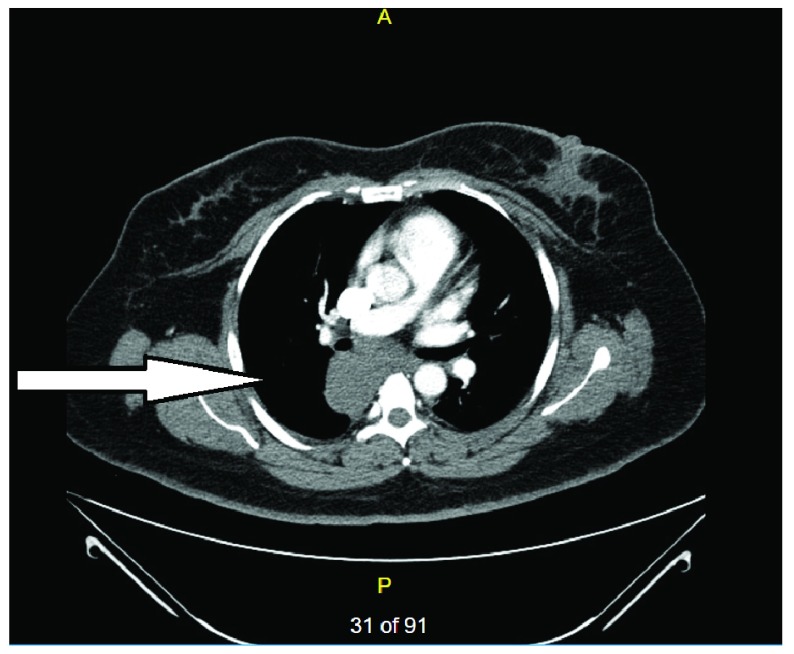
CT abdomen with contrast with chest window showing right subcarinal mass suggestive of right posterior mediastinal cyst measuring 5.4 X 5.0 cm (May 2015).

A 44-year-old Hispanic female presented with a three-week history of recurrent sharp interscapular pain radiating to the mid-sternal and epigastric region associated with refractory nausea and vomiting. She underwent cholecystectomy for intermittent epigastric pain two years ago. CT abdomen at that time showed a subcarinal mass measuring 5.4 X 5.0 cm ( Figure 1). Subsequent EUS diagnosed it as a bronchogenic cyst. EBUS guided aspiration resulted in an incomplete drainage and she was discharged after partial improvement.

**Figure 2.**
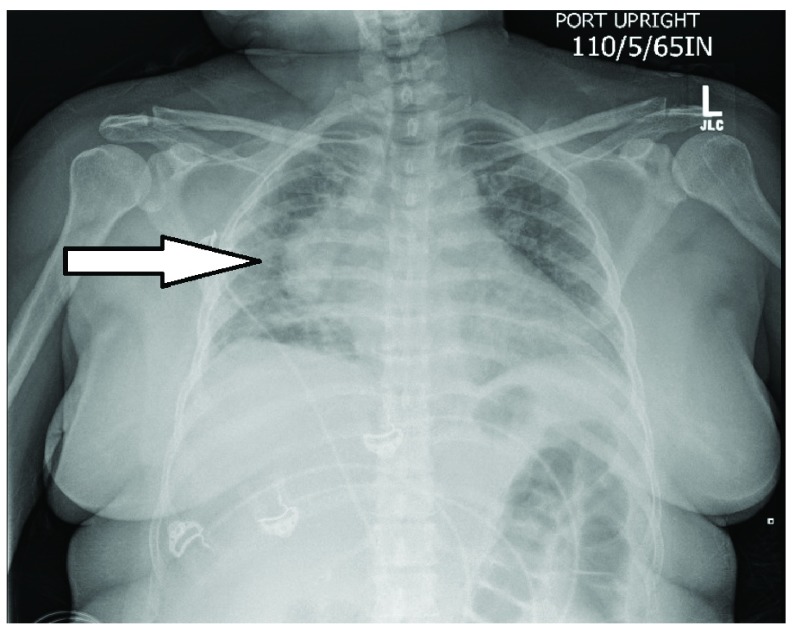
Chest X-ray showing right posterior mediastinal mass suggestive of a cyst (April 2017).

Current physical examination showed a heart rate of 126/min (normal range: 60–100/min) and respiratory rate of 20/min (normal range: 12–20/min). Initial labs showed white cell count of 10.58X10 ^3^/uL (normal range: 4000–11X10 ^3^uL), elevated inflammatory markers [ESR of 63mm/hr (normal range: 0–20 mm/hr); CRP of 116 mg/L (normal range: <3.0 mg/L)], and hypokalemic metabolic alkalosis. Electrocardiogram showed non-specific T wave changes. Chest X-ray showed right posterior mediastinal mass ( Figure 2).

**Figure 3.**
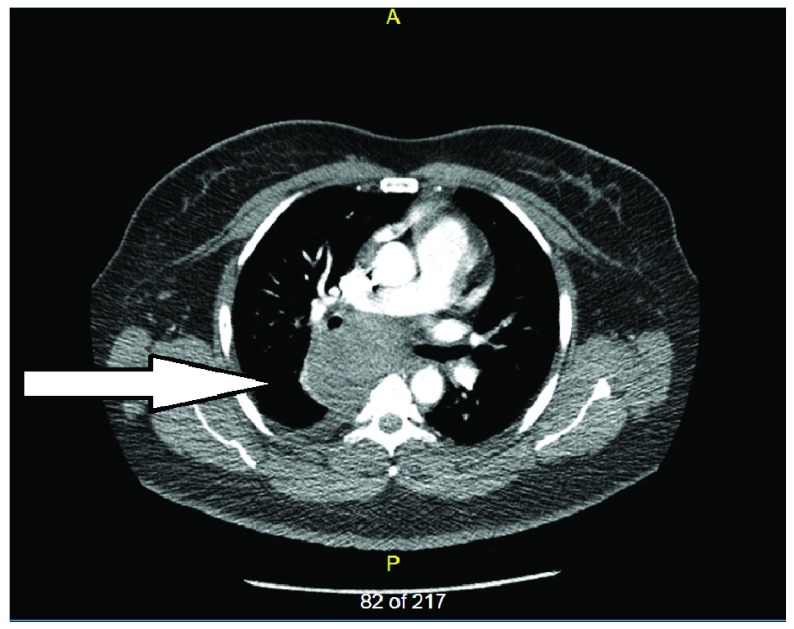
CT Chest with contrast showing right posterior mediastinal cyst measuring 9.64 X 7.7 cm (April 2017).

CT chest showed an increase in the size of the bronchogenic cyst (9.64 X 7.7 cm) with small right pleural effusion ( Figure 3).

The X-ray and CT findings were consistent with partial cyst rupture or an infected cyst. X-ray esophagogram ruled out esophageal compression or contrast extravasation. The patient’s symptoms were refractory to conservative analgesic and antiemetic measure like Dilaudid (hydromorphone) 1 mg IV every 3 hourly and Zofran (Ondansetron) 4 mg IV every 4 hourly for pain and nausea/vomiting respectively. Cardiothoracic surgery was consulted and the patient underwent right thoracotomy and surgical cyst excision. Cyst pathology was consistent with severe inflammatory changes. Within 24–48 hours after the surgery, the resolution in the patient’s symptoms were noted in terms of decrease in need of pain and nausea medications. Repeated labs showed resolution of leukocytosis.

## Discussion

Bronchogenic cysts are the rare benign congenital malformation resulting from the anomalous budding of ventral foregut and tracheobronchial tree ^1^. They are part of the bronchopulmonary foregut malformations. They are more commonly found in the mediastinum in the paratracheal and subcarinal regions. Less commonly they are found in the lung parenchyma.

Bronchogenic cyst may present with unusual symptoms posing a diagnostic challenge. Signs and symptoms of bronchogenic cyst mainly depend upon its location, size, and compression of surrounding structures like esophagus, trachea, and bronchus ^7^. Most common presentation in adult patients includes chest pain, cough, dyspnea and dysphagia ^8^.

Our patient presented with unusual symptoms of severe backache, epigastric discomfort, refractory nausea, and vomiting. It is believed that the back pain is caused by stretching of the nerves supplying the parietal pleura while the epigastric distress is caused by the stimulation of the vagal nerve ^4, 9^.

In our case, repeat CT chest confirmed an increase in the size of a bronchogenic cyst with small right pleural effusion. Considering that approximately 10% of the patients develop respiratory problems due to tracheal or bronchial compression, we performed X-ray esophagogram and ruled out esophageal compression or contrast extravasation.

At present management of symptomatic bronchogenic cyst is surgical as discussed. Management of asymptomatic cyst is controversial. It has been suggested that as most of the cysts eventually cause some symptoms or serious complications like respiratory distress from airway compression, infection and airway fistulae, surgical resection in asymptomatic patients is recommended. Also, postoperative surgical complications are more common in patients with symptomatic cysts as compared to asymptomatic cysts further implying the benefits of surgical resection of asymptomatic cysts ^10^.

## Conclusion

This case highlights the importance of recognizing bronchogenic cyst as a cause of severe back pain, refractory nausea, and vomiting. Back pain is caused by stretching of nerves supplying the parietal pleura; while nausea is caused by stimulation of vagus nerve. Prompt surgical excision can lead to complete symptom resolution and avoidance of future complications.

## Consent

Written informed consent was obtained from the patient for the publication of this case report and any accompanying images.

## Data availability

All data underlying the results are available as part of the article and no additional source data are required.
