# Supplementary material for: Case Report: Severe back pain, epigastric distress and refractory nausea; an unusual presentation of mediastinal bronchogenic cyst
Source: F1000Res. 2018 Jun 28;7:960. [Version 1] doi: 10.12688/f1000research.15128.1 (PMC6171728; doi:10.12688/f1000research.15128.1)
Supplement: Peer review history

# Peer review: Referee response for version 1

**DOI:** 10.5256/f1000research.16481.r37985

**Published:** 2018-09-20

**Authors:** Ali Dahhan

**Affiliations:** 1 University of Louisville, Louisville, Louisville, KY, USA

This is a case report of bronchogenic cyst. It highlights the importance of imaging, including computed tomography. The article is well-written. One minor suggestion is to consider adjusting the position of the arrows in Figures 1 and 3 as it points at the right lung rather than the cyst - instead an asterix over the cyst can also be practical without hiding parts of the figures.

I have read this submission. I believe that I have an appropriate level of expertise to confirm that it is of an acceptable scientific standard.

# Peer review: Referee response for version 1

**DOI:** 10.5256/f1000research.16481.r37054

**Published:** 2018-09-19

**Authors:** Dipak K. Mukherjee

**Affiliations:** 1 Department of Respiratory Medicine, Basildon and Thurrock University Hospital, Basildon, UK

Bronchogenic cyst diagnosis on EBUS and complication of Infection.

 Needs to alert that Needling with EBUS needle is a recognised complication and some practitioners caution against using EBUS needle if diagnosis can be secured by other means but sometime if there is uncertainty about the diagnosis one has to do the EBUS needling.

 One has to understand the potential chance of recurrence in absence of complete removal of cyst wall after surgery and possibly need mentioning.

 On the whole the article is well written.

 similar article we presented: Mogal et al ^1^

I have read this submission. I believe that I have an appropriate level of expertise to confirm that it is of an acceptable scientific standard.

## References

[1] MogalRBanerjeeNYungBMukherjeeD: A Young Woman With Severe Chest Pain After Undergoing Endobronchial Ultrasound-guided Transbronchial Needle Aspiration for a Large Mediastinal Mass. *J Bronchology Interv Pulmonol*.2016;23(3) : 10.1097/LBR.0000000000000224 236-8 10.1097/LBR.0000000000000224 27070336
